# Fielding the research participant perception survey to evaluate a culturally tailored Latinx cohort study

**DOI:** 10.1017/cts.2024.629

**Published:** 2024-10-29

**Authors:** Sierra Lindo, Jamie Roberts, James Goodrich, Alejandra Mella-Velazquez, Michael D. Musty, Alex C. Cheng, Rhonda G. Kost, Rosa M. Gonzalez-Guarda, Ranee Chatterjee

**Affiliations:** 1 Duke Clinical and Translational Science Institute, Recruitment Innovation Center, Duke University, Durham, NC, USA; 2 Duke Cancer Institute, Duke University, Durham, NC, USA; 3 Duke Psychiatry & Behavioral Sciences, Duke University, Durham, NC, USA; 4 Trinity College of Arts and Sciences, Duke University, Durham, NC, USA; 5 Department of Biomedical Informatics, Vanderbilt University, Nashville, TN, USA; 6 Center for Clinical and Translational Science, The Rockefeller University, New York, NY, USA; 7 Duke University School of Nursing, Durham, NC, USA; 8 Duke University School of Medicine, Durham, NC, USA

**Keywords:** Latinx population, Hispanic population, research participation, research experiences, satisfaction survey

## Abstract

**Introduction:**

Latinx populations are underrepresented in clinical research. Asking Latinx research participants about their research experiences, barriers, and facilitators could help to improve research participation for these populations.

**Methods:**

The Salud Estres y Resilencia (SER) Hispano cohort study is a longitudinal cohort study of young adult Latinx immigrants whose design and conduct were tailored for their study population. We administered the Research Participant Perception Survey (RPPS) to SER Hispano participants to assess their experiences in the study. We describe overall results from the RPPS and compare results of surveys administered to SER Hispano participants via email versus telephone.

**Results:**

Of 340 participants who were contacted with the RPPS, 142 (42%) responded. Among respondents, 53 (37%) responded by initial email contact; and 89 (63%) responded by subsequent phone contact. The majority of respondents were between 35 and 44 years of age (54%), female (76%), and of Cuban origin (50%). Overall, research participants expressed high satisfaction with their research experience; 84% stated that they would “definitely” recommend research participation to friends and family, with no significant difference by method of survey administration (*P* = 0.45). The most common factor that was chosen that would influence future research participation was having summary results of the research shared with them (72%).

**Conclusion:**

We found that culturally tailored studies can be good experiences for Latinx research participants; and we found that use of the RPPS can be administered successfully, particularly when administered by more than one method, including telephone, to evaluate and to improve research experiences for this population.

## Introduction

The Latinx population in the USA is the nation’s largest racial minority group, yet health disparities persist within this community [1]. It is essential that healthcare advances are made to improve this population’s health and to reduce disparities. Improvements in healthcare delivery and outcomes necessitate clinical research to advance the science of health; and clinical research requires equitable participation by different populations, based on age, sex, gender, race, and ethnicity to ensure generalizability of findings and/or to determine if different populations have different responses to or needs for health care, all with the goal of reducing health disparities. The National Institutes of Health (NIH) Revitalization Act of 1993 established guidelines to ensure inclusion of women and minority populations in clinical research to achieve this goal. Since then, while inclusion of women and certain race groups has improved, the reporting of and the inclusion of Latinx populations in clinical research in the USA continue to lag, and in fact, may have decreased since the passing of the Revitalization Act [2–4].

Prior studies have examined the barriers and facilitators to participation in clinical research by Latinx populations [5]. Barriers include the costs and time required for participation. Facilitators include trust in the research team, language concordance, and family support. These studies of facilitators and barriers are generally based on intervention studies designed to test specific tactics to improve recruitment or retention of underrepresented populations, including Latinx populations, while fewer studies are observational and designed to report the perceptions and attitudes of Latinx populations related to research more generally [5]. Ongoing assessments and improvements in research processes and inclusivity are needed, particularly for Latinx populations, to enhance their experiences and ultimate trust in clinical research.

The Research Participants’ Perceptions Survey (RPPS) is a validated tool developed to capture key aspects throughout the experience of research participation in order to assess how research processes are being received by participants and to understand potential areas of improvement [6,7]. The validation of the RPPS included Latinx participants. The RPPS is available in English and Spanish [7], can be administered at the health system or individual study level, and survey findings can be used to tailor research programs for different populations. The RPPS is an evaluation tool that can be used to guide and evaluate efforts to improve the research participation experience, including those from populations that are underrepresented in clinical research.

The Salud Estres y Resilencia (SER) (Health Stress and Resilience) Hispano study is a longitudinal community-engaged research study that was developed in order to guide interventions that may help reduce health disparities among the Latinx community [8,9]. This study was designed collaboratively by an academic research team and a community-based organization to reduce some of the known barriers of research participation for a cohort of young adult Latinx immigrants. Some of the tailored features of the SER Hispano study were to include researchers and research staff who were bicultural and bilingual as well as careful attention and effort towards the translation and acculturation of study-related materials in both English and Spanish.

Towards the end of the SER Hispano study and in collaboration with a project to help institutions streamline RPPS administration, the Empowering the Participant Voice (EPV) project [10], we administered the RPPS to SER Hispano study participants to gather information on their research experiences to help determine if the design and conduct of the study were successful in creating a positive research participation environment for its participants and to identify additional measures that could improve the experience and recruitment of the Latinx population, a population historically underrepresented in research, in future research studies. Here, we describe the administration and results of the RPPS from the SER Hispano study cohort.

## Materials and methods

### Research participant perceptions survey

The RPPS was developed in 2012 through a multi-institutional collaboration, using mixed methods and a participant-centered approach [6,7]. The survey assesses key aspects of the research experience as identified by participants and validated through psychometric testing. The RPPS examines the participant experience across the life of a study and was developed to collect measures that could be used to drive evidence-based improvements in research processes, participant experiences, and overall satisfaction for research participants [6,7]. After initial validation of a long version, subsequent shorter survey versions were developed and validated [11]. For the purposes of this study, we utilized the RPPS-Short survey (RPPS-S) instrument. The RPPS-S version used in the EPV project is made up of six questions that collect characteristics of the respondent (age, sex, gender, race, ethnicity, education level); two questions that assess the experience broadly, with a 0 (worst) to 10 (best) numerical scale to rate the overall experience and a Likert rating of whether they would recommend research participation to friends and family; actionable questions, including six key questions that, during validation, were shown together to account for 96% of the variance in the Overall Rating score, including questions related to informed consent, being listened to, being treated with courtesy and respect, and knowing how to reach study staff [11,12]; additional questions related to the study experience; and a question related to future research participation. All survey versions, including the one administered as part of the EPV project, include an open text field at the end of the survey to collect additional comments from the respondent.

### Description of the SER Hispano cohort study

The SER Hispano study is a longitudinal community-engaged cohort study of 391 young adult (ages 18–44 at the time of recruitment) Latinx immigrants in North Carolina whose predominant language was Spanish [8,9]. The study excluded non-Spanish-speaking Latinxs, e.g. Brazilians or any indigenous Latinx populations that do not speak Spanish. The cohort was followed over two years and focused on studying the effects of acculturation stress, resilience, stress biomarkers, and physical, behavioral, and mental health outcomes [9].

Participants in this cohort study were notified to expect to receive a link to the RPPS-S survey through the quarterly SER Hispano newsletter that they received as part of study participation, either at in-person visits or via email for those who agreed to this method of contact. Survey notification in the newsletter was provided in English and Spanish. Along with the newsletter content, flyers were provided to those participants who had remaining in-person visits, alerting them of what to expect when completing the survey (e.g. examples of questions included in the survey).

### Survey administration

The RPPS-S was distributed to 340 participants who agreed to be contacted for participation after the SER Hispano study procedures were completed. The survey was initially sent via email with a link to complete the survey in REDCap. The email, which was sent out in Spanish, included an invitation for all participants to complete a brief survey that would gather information about their experience participating in the study. The email invitation contained specific mention of the SER Hispano study and the name of its principal investigator to ensure that participants had a clear understanding of the research study for which they were completing the survey. Finally, the email included a link directing the participant to REDCap to complete the survey in English or Spanish. The initial email invitation to complete the survey was followed by two subsequent reminders, each a week apart, if the survey was not yet completed.

Those participants who did not complete the survey online after the initial email and reminders, and who had agreed to be contacted by phone, were subsequently contacted by phone (two attempts) to complete the survey. Administration of the survey via telephone call was conducted by a research staff member who was not involved in either the SER Hispano study or the EPV project. Responses given to the survey over the phone were entered into the REDCap database by the phone survey administrator.

### Statistical analyses

We describe baseline characteristics of our cohorts of interest with frequencies. In addition, we calculate response rates to the RPPS-S questions overall and also response rates by email or telephone. The answers to survey questions are scored by the frequency of the optimal answer (Top Box scores) [7] and also by the frequencies of all responses. Responses to the main survey questions were compared between those who responded via email to those who responded by telephone using Fisher’s exact tests, assuming an alpha level of 0.01. Such a comparison was not conducted for the question related to future research participation or the free text response question.

## Results

Of the 340 SER Hispano study participants who agreed to be contacted, 142 responded either by telephone or email, yielding an overall response rate of 42%. Of those that responded, 53 (37%) completed it via email and, through subsequent contact with those that did not respond via email, an additional 89 (63%) completed the survey via telephone. Of those completing the survey via the emailed REDCap link, 26 (49%) completed the survey in English; of those who completed the surveys by telephone, all chose to have the survey administered in Spanish.

Selected baseline characteristics of the SER Hispano participants at the time of the RPPS-S administration and of the SER Hispano participants who responded to the survey are described in Table 1; and demonstrated that the survey respondents were representative of the overall study cohort. Participants’ ages ranged between 18 and 54, with the majority (54%) being between 35 and 44 years of age and with the majority of participants being female (76%).

**Table 1.** Selected baseline characteristics of Salud Estres y Resilencia (SER) Hispano and research participant perceptions survey (RPPS) participants

Characteristics	142 RPPS Responders	340 SER Hispano willing to be contacted
**Age (years)**		
18–34	47 (33.1%)	127 (37.4%)
35–44	76 (53.5%)	167 (49.1%)
45–54	18 (12.7%)	45 (13.2%)
**What is your sex?**		
Female	108 (76.1%)	234 (68.8%)
Male	33 (23.2%)	106 (31.2%)
Intersex	0	–
**How would you describe your gender identity?**		
Female (including transgender women)	102 (71.8%)	236 (69.4%)
Male (including transgender men)	32 (22.5%)	104 (30.6%)
Non-binary, gender-fluid, agender	1 (0.7%)	–
Prefer not to say	5 (3.5%)	–

[-] Questions not asked in SER Hispano study, so no responses available.

Most participants identified with their country of origin, the majority being of Cuban origin (50%), followed by Other (46%). Most participants did not identify with a specific racial group and chose to only respond to the ethnicity question via both email and telephone. For those who identified as Latinx and also responded to the race question (*n* = 37), most (*n* = 32) identified as White. The question asking “*What is the highest grade or level of school that you have completed*?” was met with confusion from phone participants about the categorization of different educational levels. For phone participants, the survey administrator translated the question in Spanish as “*How many years were you in school for*?” and then converted the number of school years stated into the equivalent US educational grade level. More respondents (26%) indicated that the highest level of education achieved was high school graduate/GED or equivalent, 18% had more than a 4-year college degree, 18% had a 4-year college degree, 14% had some college education, 13% had some high school education, and 10% had 8th grade or less.

Responses to the RPPS-S questions are shown in Table 2. Participants were asked two questions to assess their overall experience in the research study, and from their responses, we found that participants generally had a very good experience, and we found no significant differences in responses via email versus telephone. When asked to use a scale to rate their overall experience in the research study, 0 being the worst possible experience and 10 being the best, 80% of participants responded with either a 9 or 10 (the defined Top Box score), with no significant difference in response by method of survey administration (*P* = 0.67). When answering the questio*n “Would you recommend joining a research study to your family and friends?”* most respondents (98%) selected either “*definitely yes” (84%)* or “*probably yes” (14%)*, again with no significant difference in response by survey administration (*P* = 0.45).

**Table 2.** Research participant perceptions survey (RPPS) responses from Salud Estres y Resilencia (SER) Hispano study participants

RPPS Question	Overall Responses *n* = 142	Email Responses *n* = 53	Telephone Responses *n* = 89
**Would you recommend joining a research study to your family and friends?**			
Definitely yes*	119 (83.8%)	42 (79.2%)	77 (86.5%)
Probably yes	20 (14.1%)	10 (18.9%)	10 (11.2%)
Probably no	1 (0.7%)	0 (0%)	1 (1.1%)
Definitely no	2 (1.4%)	1 (1.9%)	1 (1.1%)
**Please use the scale below to rate your overall experience in the research study, where 0 is the worst possible experience, and 10 is the best possible experience.**			
10 (best)*	80 (56.3%)	29 (54.7%)	51 (57.3%)
9*	34 (23.9%)	13 (24.5%)	21 (23.6%)
8	22 (15.5%)	10 (18.9%)	12 (13.5%)
7	6 (4.2%)	1 (1.9%)	5 (5.6%)
**Did the Informed consent form prepare you for what to expect during the study?**			
Yes-completely*	81 (57.0%)	24 (45.3%)	57 (64.0%)
Yes-mostly	39 (27.5%)	22 (41.5%)	17 (19.1%)
Yes-somewhat	13 (9.2%)	5 (9.4%)	8 (9.0%)
No	4 (2.8%)	2 (3.8%)	2 (2.2%)
**Did the information and discussions you had before participating in the research study prepare you for your experience in the study?**			
Yes-completely*	77 (54.2%)	26 (49.1%)	51 (57.3%)
Yes-mostly	36 (25.4%)	18 (34.0%)	18 (20.2%)
Yes-somewhat	15 (10.6%)	7 (13.2%)	8 (9.0%)
No	10 (7.0%)	2 (3.8%)	8 (9.0%)
**Did the research team members listen carefully to you?**			
Always*	131 (92.3%)	47 (88.7%)	84 (94.4%)
Usually	10 (7.0%)	6 (11.3%)	4 (4.5%)
Sometimes	1 (0.7%)	0 (0%)	1 (1.1%)
Never	0 (0%)	0 (0%)	0 (0%)
**Did the research team members treat you with courtesy and respect?**			
Always*	139 (97.9%)	51 (96.2%)	88 (98.9%)
Usually	2 (1.4%)	1 (1.9%)	1 (1.1%)
Sometimes	1 (0.7%)	1 (1.9%)	0 (0%)
Never	0 (0%)	0 (0%)	0 (0%)
**During your discussion about the study, did you feel pressure from the research staff to join the study?**			
Never*	136 (95.8%)	48 (90.6%)	88 (98.9%)
Sometimes	1 (0.7%)	1 (1.9%)	0 (0%)
Usually	1 (0.7%)	1 (1.9%)	0 (0%)
Always	4 (2.8%)	3 (5.7%)	1 (1.1%)
**Did the research staff do everything possible to provide assistance with any language difference you might have?**			
Always*	40 (28.2%)	21 (39.6%)	19 (21.3%)
Usually	0 (0%)	0 (0%)	0 (0%)
Sometimes	1 (0.7%)	0 (0%)	1 (1.1%)
Never	13 (9.2%)	5 (9.4%)	8 (9.0%)
No language difference	88 (62.0%)	27 (50.9%)	61 (68.5%)
**When you were not at the research site did you know how to reach the research team if you had a question?**^**1**^			
Always*	95 (66.9%)	27 (50.9%)	68 (76.4%)
Usually	33 (23.2%)	20 (37.7%)	13 (14.6%)
Sometimes	5 (3.5%)	3 (5.7%)	2 (2.2%)
Never	7 (4.9%)	3 (5.7%)	4 (4.5%)
**When you were not at the research site and you needed to reach a member of the research team, were you able to reach him/her as soon as you wanted?**^**1**^			
Always*	86 (60.6%)	21 (39.6%)	65 (73.0%)
Usually	14 (9.9%)	11 (20.8%)	3 (3.4%)
Sometimes	8 (5.6%)	4 (7.5%)	4 (4.5%)
Never	4 (2.8%)	2 (3.8%)	2 (2.2%)
Did not need to reach the research team	28 (19.7%)	15 (28.3%)	13 (14.6%)
**Did you feel you were a valued partner in the research process?**			
Always*	124 (87.3%)	45 (84.9%)	79 (88.8%)
Usually	14 (9.9%)	6 (11.3%)	8 (9.0%)
Sometimes	1 (0.7%)	1 (1.9%)	0 (0%)
Never	2 (1.4%)	1 (1.9%)	1 (1.1%)
**If you considered leaving the study, did you feel pressure from the Research Team to stay?**			
Never*	80 (56.3%)	27 (50.9%)	53 (59.6%)
Sometimes	2 (1.4%)	0 (0%)	2 (2.2%)
Usually	0 (0%)	0 (0%)	0 (0%)
Always	0 (0%)	0 (0%)	0 (0%)
Did not consider leaving the study	57 (40.1%)	24 (45.3%)	33 (37.1%)
**Did the research staff respect your cultural background (e.g. language, religion, ethnic group)?**			
Always*	97 (68.3%)	36 (67.9%)	61 (68.5%)
Usually	0 (0%)	0 (0%)	0 (0%)
Sometimes	0 (0%)	0 (0%)	0 (0%)
Never	2 (1.4%)	1 (1.9%)	1 (1.1%)
No cultural issues	42 (29.6%)	15 (28.3%)	27 (30.3%)
**Did you have enough physical privacy while you were in the study?**			
Always*	134 (94.4%)	48 (90.6%)	86 (96.6%)
Usually	4 (2.8%)	3 (5.7%)	1 (1.1%)
Sometimes	0 (0%)	0 (0%)	0 (0%)
Never	2 (1.4%)	0 (0%)	2 (2.2%)
**Did the study require that you already have a disease or condition in order to enrol?**			
Yes	5 (3.5%)	2 (3.8%)	3 (3.4%)
No	136 (95.8%)	50 (94.3%)	86 (96.6%)
**Did the study involve taking a drug or a supplement or the use of a new medical device, or undergoing a new medical procedure?**			
Yes	1 (0.7%)	0 (0%)	1 (1.1%)
No	137 (96.5%)	50 (94.3%)	87 (97.8%)
Not sure	4 (2.8%)	3 (5.7%)	1 (1.1%)
**How much did the study demand of you? (Pick the answer that most closely describes your experience)**^**1**^			
Simple (e.g. a few visits or simple tests or surveys)	80 (56.3%)	44 (83.0%)	36 (40.4%)
Moderate (e.g. multiple visits or a short inpatient stay; only a few procedures, not risky or intense)	59 (41.5%)	9 (17.0%)	50 (56.2%)
Intense (e.g. long or multiple inpatient stays or many visits; procedure(s) that are intense, risky, or complex)	3 (2.1%)	0 (0%)	3 (3.4%)
**Before you joined the study, how did the study team discuss the details of the study with you?**			
Mostly through the email or video or telephone conversations	24 (16.9%)	9 (17.0%)	15 (16.9%)
Mostly while physically in the same place with a member of the study team	50 (35.2%)	22 (41.5%)	28 (31.5%)
A mix of conversations taking place both physically in the same place and over telephone/video/computer	58 (40.8%)	20 (37.7%)	38 (42.7%)
No discussion with the study team before joining the study	2 (1.4%)	0 (0%)	2 (2.2%)
I do not remember	8 (5.6%)	2 (3.8%)	6 (6.7%)

1 Fisher’s exact test of significance, *P* < 0.01.

* Considered as “Top Box” response for question.

For the actionable questions, survey respondents overall expressed positive experiences, with no significant differences in responses by survey administration for questions related to informed consent, respect, privacy, cultural and language differences. When asked if they knew how to reach the research team when participants were not on site and if they were able to reach the research team when they wanted, significantly more phone respondents gave their experiences the highest rating compared to email respondents (*P*<0.01 for both questions).

For the other questions related to their study experiences, there were generally no differences in responses by survey administration method. However, more participants described the demands of the study as being “*moderate*” or “*intense*” when answering by phone rather than “*simple*,” which was the more common answer for those responding by email (*P* < 0.01).

**Figure 1.**
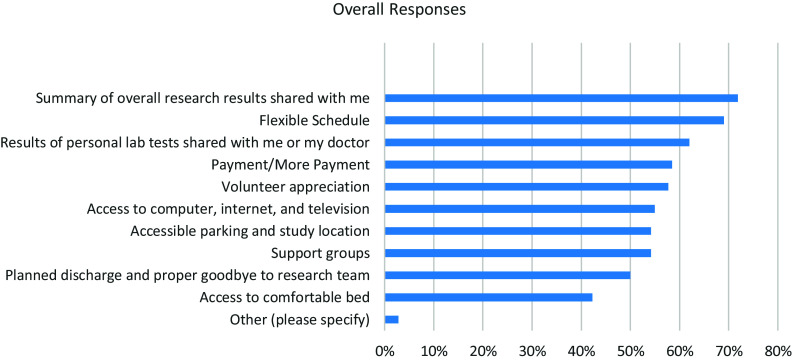
Factors influencing future research participation^1^. ^1^ Responses from 142 Salud Estres y Resilencia (SER) Hispano study participants to the question: “which of the following things would be important for you in a future study? Please check all that apply.”

As part of the RPPS-S, respondents were asked to select what factors would most influence their participation in a future research study (Figure 1). Of the 142 respondents, 72% selected “*Summary of overall research results shared with me*” 69% chose “*flexible schedule*;” and 62% chose “*results of personal lab tests shared with me or my doctor*.”

Finally, survey respondents had the opportunity to respond to a free text option to share anything additional about their experience in the study. There were 50 free text comments submitted. The main themes of the comments included requests to receive the results of the study (either aggregate or personal) and requests to be contacted to participate in future studies.

### Lessons learned from administering survey by phone

Survey participants who were contacted by phone immediately wanted to know the duration of the survey before agreeing to participate. Surveys conducted over the phone were found to take between 5 to 7 minutes to complete. Multiple choice and check-all-that-apply questions required repetition by the survey administrator, as many participants would forget the original question that was being asked and the provided answer choices. Yes/no questions were found to require less repetition for participants and the survey administrator. Some questions were perceived as open-ended questions and gave participants room to elaborate, which led to some difficulty for the survey administrator to categorize their response with an answer choice. This was a limitation to fielding the survey by telephone, as there were no plans in place to capture open-ended responses verbatim.

## Discussion

In this study, we describe the methods and results of administering the validated Research Participants’ Perceptions Survey-Short (RPPS-S) to Latinx immigrant research participants enrolled in a longitudinal cohort study, the SER Hispano study, which was specially designed to overcome many of the barriers that this population faces related to research participation. The involvement in clinical research of Latinx populations is currently low. People of Latinx ethnicity currently make up over 19% of the US population ; however, currently in the USA, only 11% of trial participants are of Latinx ethnicity, with many studies recruiting far less [5]. While efforts have been made to increase Latinx representation in clinical research, including the 1993 NIH Revitalization Act, in certain measures, participation in clinical research among this population has actually decreased [2]. Identifying potential barriers and facilitators to clinical research participation for Latinx populations and then addressing these to encourage participation are essential for advancement of health discoveries. Recognizing the importance of research participation by Latinx populations, in its study design and conduct, the SER Hispano study attempted to address known barriers for this population; the RPPS-S survey was administered to SER Hispano study participants to help determine if these attempts were successful.

Administration of the RPPS-S to the SER Hispano Latinx immigrant cohort posed some unique considerations which had not been previously described in the use of this survey. Investigators leading the SER Hispano study anticipated that distribution of the survey via email might lead to a lower response rate compared to other populations. Prior studies of the RPPS, primarily distributed via traditional mail, describe a response rate of about 30% overall in populations of mixed race/ethnicity with about 5% of those study populations being of Latinx ethnicity [12,13]. Comparison of the administration of the survey via different methods has been previously examined at a single institution with a mixed population of races (21% of Black race; 4% of Hispanic ethnicity) [14]. That study found that response rates were also approximately 30% when distributed via traditional mail, electronic health record’s patient portal, or when administered over the phone, but that email distribution led to a lower response rate of about 15% [14]. In that study, 33 participants were of Latinx ethnicity and were sent the survey by one of the 4 methods; 7% of these participants receiving the survey by mail responded, while 30% of those that were called responded to the survey over phone [14]. In our study of the SER Hispano study cohort, we had similar response rates; email distribution of a REDCap link to the survey resulted in a 16% response rate, while telephone administration of the survey resulted in a 26% response rate.

The experiences among this research participant cohort of Latinx immigrants in the SER Hispano study were overall very positive. Of RPPS-S respondents, 80% gave a Top box score for Overall Rating of their research experience (ratings of 9 and 10), which is higher than the Overall Rating scores from participants of all races/ethnicities in other studies at our institution which was 68% (*unpublished data*). The SER Hispano participants also rated their Overall Experiences higher than the mean score for responses aggregated by the EPV consortium, but similar to responses from Latinx participants in the EPV consortium database (78% Top Box score) (*manuscript in preparation*). Moreover, 84% stated that they would “definitely” recommend research participation to friends and family, which is higher than responses given by participants of other studies at our institution overall (60%) (*unpublished data*). This positive response rate is also higher than response rates from the EPV consortium database overall (61%) and from the Latinx participants of the EPV consortium database (67%) (*manuscript in preparation*). The SER Hispano study was designed with the goal to overcome barriers that Latinx populations often face, with a research team which was comprised of study staff of diverse backgrounds, who were Spanish-speaking, with study documents which were carefully translated into Spanish, taking into account content and context, and with study procedures that were developed with and for the community that they were studying [8,9]. Based on the results of the RPPS-S of the participants of the SER Hispano study, it is evident that the study design and conduct were successful in making the research experience a good one for this population; and study teams planning to work with similar populations could use similar methods.

The RPPS-S was administered in two ways to the participants of the SER Hispano study, via email as a REDCap link, with the option of completing the survey in either English or Spanish; as well as via telephone, with a Spanish-speaking staff member. The preferred language was Spanish for all participants who were contacted by phone and for almost half of the participants who responded by email, which highlights the importance of offering research-related documents in languages other than English for participants whose first language is not English. The process of translation must take into account not only literal meanings but also cultural and linguistic meanings, depending on the population being studied [8]. An example of this is the question that inquired about highest grade or level of school completed which was met with confusion from phone participants, possibly due to the majority of participants’ education being attained outside of the USA. The RPPS has been translated into neutral broadcast Spanish by a team of professional translators at the University of Texas Southwestern [7] and may benefit from review when administered to different populations.

There was some concern that there might be differences in responses for this population by the method of survey administration, with a particular concern for a social desirability bias when administered via telephone [15–17]. However, we found that for the majority of questions, there were no statistically significant differences in responses when administered via email or via telephone. With both methods, participants expressed high satisfaction with their research experience and expressed that they would encourage others to participate in research. With both methods of administration of the survey, high proportions of participants expressed being treated with courtesy and respect, being listened carefully to, and expressed that there were no language differences. However, there were some statistically significant differences in responses by method of administration for certain questions. For the question “*How much did the study demand of you?,*” more of those that responded by telephone expressed that the study had moderate to intense demands, while the majority of those that responded by email expressed that the study was relatively simple. And, for the questions related to being able to contact study staff when they were not at study procedures, more of the participants who answered by phone expressed that they were “always” or “usually” able to reach study staff compared to those responding via email, and more of the participants who responded by email compared to those answering by phone said that they did not need to reach the study staff when not at the research site. These questions, particularly those related to being able to reach study staff, could potentially reflect some social desirability bias, perhaps, in order to benefit the research staff.

When asked about factors that would influence their decision to participate in future studies, the most frequently chosen factor for this study population of Latinx immigrants was having the overall results of the research shared with them. Additionally, in the free text response question, participants reiterated the importance of receiving results from studies. This finding is similar to that found in studies of the administration of the RPPS survey to research participants from other academic centers, as well as from other surveys of research participants [12,13,18] (and *manuscript in preparation*). While the Final Rule mandates that clinical trials do have to register and share results on clinicaltrials.gov [19], there is no current mandate that the summary of research study results has to be shared directly with the research participants in plain language or in the primary language of the participant. Sharing of summary results of any research study with their study participants would be a way to improve the research experience for all populations, including Latinx populations.

This study demonstrates that when a research study, such as the SER Hispano study, is designed thoughtfully, in collaboration with the population being studied, research participation by a Latinx population can be successful and a good experience for them; and this study demonstrates that this positive experience can be captured by the RPPS-S. There are limitations of this study, however, which include the fact that this is a single study, which limits the generalizability of the findings. Within this single study, the study participants had differences in years lived within the USA and differences in countries of origin, as well as differences in other factors which could influence their experiences, and whose impact on responses was not analyzed. In addition, within the SER Hispano study, not all participants responded to the survey, and it is possible that some participants did have negative experiences or experiences which were not captured. However, through the administration of the survey by both email and telephone, we were able to get responses from a higher proportion of participants than in prior studies of the RPPS survey, which have generally administered the survey using only one method. Finally, in our statistical reporting of differences in responses to the RPPS-S by email or telephone, we did not adjust for multiple comparisons; however, we did choose a more stringent cutoff for the *P*-value of significance at 0.01 to help account for this.

In this study, we describe the research experiences of participants in a cohort study of Latinx immigrants as measured by the RPPS-S survey. We found that to optimize response rate, more than one method of survey administration, including telephone administration, was helpful; and we found that responses generally did not differ by method of administration. We found that research participation of Latinx populations can be successful and can be a positive experience for them, particularly when the study is designed in ways that accommodate their cultural and language needs. Additionally, we found that sharing the results of the research with participants is highly important to them and could demonstrate the value of their participation, improve trust, and encourage future participation.
